# Diagnostic challenges and potential early indicators of breast periprosthetic anaplastic large cell lymphoma: A case report

**DOI:** 10.1097/MD.0000000000021095

**Published:** 2020-07-24

**Authors:** Daniele La Forgia, Annamaria Catino, Alfonso Fausto, Daniela Cutrignelli, Annarita Fanizzi, Gianluca Gatta, Liliana Losurdo, Arianna Maiorella, Marco Moschetta, Cosmo Ressa, Anna Scattone, Aurelio Portincasa

**Affiliations:** a I.R.C.C.S. Istituto Tumori “Giovanni Paolo II”, Bari; b Dip. di Diagnostica per Immagini, Azienda Ospedaliera Universitaria Senese, Siena; c Dip. di Medicina di Precisione, Università degli Studi della Campania Luigi Vanvitelli, Napoli; d Dip. di Scienze Fisiche, della Terra e dell’Ambiente, Università degli Studi di Siena, Siena; e Dip. di Emergenza e Trapianti d’organi, Università degli Studi di Bari “Aldo Moro,” Bari; f Dip. di Chirurgia Plastica, Università di Foggia, Foggia, Italy.

**Keywords:** anaplastic large cell lymphoma, breast implant, breast magnetic resonance, periprosthetic anaplastic lymphoma

## Abstract

**Rationale:**

Anaplastic large T-cell lymphoma (BI-ALCL) is a rare primitive lymphoma described in women with breast implant prostheses, which has been arousing interest in recent years due to its potentially high social impact. The difficult diagnosis associated with the high and increasing number of prosthetic implants worldwide has led to hypothesize an underestimation of the real impact of the disease among prosthesis-bearing women. The aim of this work is to search for specific radiological signs of disease linked to the chronic inflammatory pathogenetic mechanism.

**Patient concerns:**

This work describes a case of BI-ALCL in an American woman with no personal or family history of cancer, who underwent breast augmentation for esthetic purposes at our Institute. After about 10 years of relative well-being, the patient returned to our Institute with clear evidence of breast asymmetry due to the increase in volume of the right breast which had progressively become larger over a period of 6 months. There was no evidence of palpable axillary lymph nodes or other noteworthy signs.

**Diagnosis:**

The ultrasound and magnetic resonance (MR) tests indicated the presence of seroma with amorphous material in the exudate which was confirmed by indirect signs, visible in right breast mammography. Due to suspected cold seroma, an ultrasound-guided needle aspiration was performed for the cytological analysis of the effusion which highlighted the presence of a number of large-sized atypical cells with an irregular nucleus with CD30 immunoreactivity, leucocyte common antigen (CD45) compatible with the BI-ALCL diagnosis.

**Interventions:**

In our case, a capsulectomy was performed because the disease was limited inside the capsule and periprosthetic seroma. The final histological examination confirmed the stage.

**Lessons:**

The patient is being monitored and shows no signs of recurrence of disease >24 months after surgery.

**Conclusion:**

A diagnosis of BI-ALCL can be reached using new radiological indicators, such as fibrin, which is clearly visible by MR in the form of nonvascularized debris of amorphous material hypointense in all sequences, free flowing or adhered to the external surface of the prosthesis.

## 1 Introduction

Large-cell anaplastic periprosthetic T-lymphoma (BI-ALCL) is a rare disease, which represents one of the primitive breast lymphomas, first described by Keech JA^[1]^ in 1997.

The disease has an incidence of 2.03 per million inhabitants and a prevalence of 1 in 30,000 women with prosthetic implants.^[2]^ de Boer^[3]^ reports a cumulative age risk in women with prostheses: 29 per million for 50-year-old women and 82 per million for 70-year olds. Although these estimates are apparently reassuring, some authors underline the complexities of correctly defining the impact and prevalence of a rare disease in a population and the high risk of underestimation in the case of BI-ALCL due to the difficulty of early recognition and correct monitoring linked to the high number of prostheses implanted in the world, in constant and progressive growth.^[4]^

In our work, we report a case of anaplastic periprosthetic lymphoma in a woman of American origin, analyzed by means of multiple radiological methods, who underwent surgery at an early stage with the aim of carrying out a retrospective study on the early signs of the disease.

## 2 Case report

**Figure 1:**
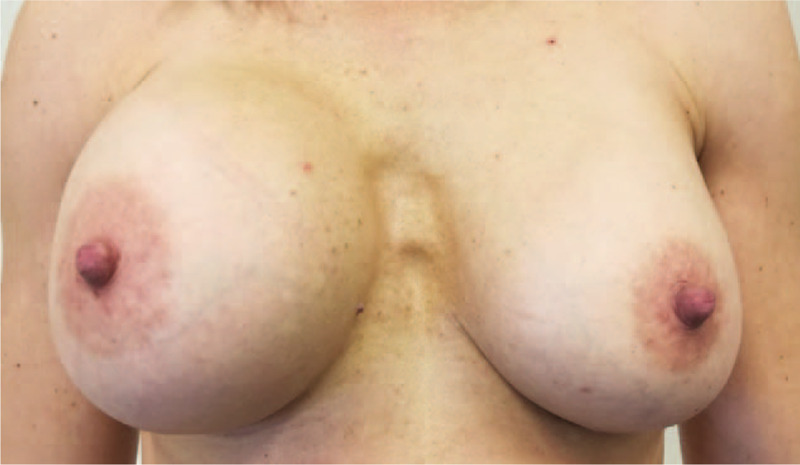
Breast asymmetry: increase in volume of the right sinus with respect to the left one.

**Figure 2:**
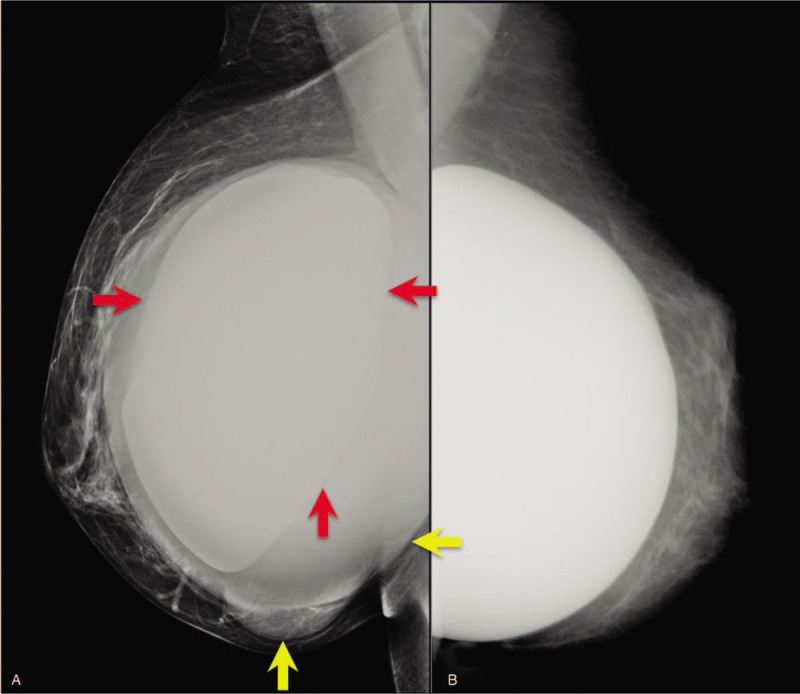
Clear clinical and radiological asymmetry of the breast by volume increase of the right sinus (A) with respect to the left one (B): the mammogram shows a radiopaque double contour consisting of the prosthesis (red arrows) and the pouring present between the prosthesis and the capsule (yellow arrows).

**Figure 3:**
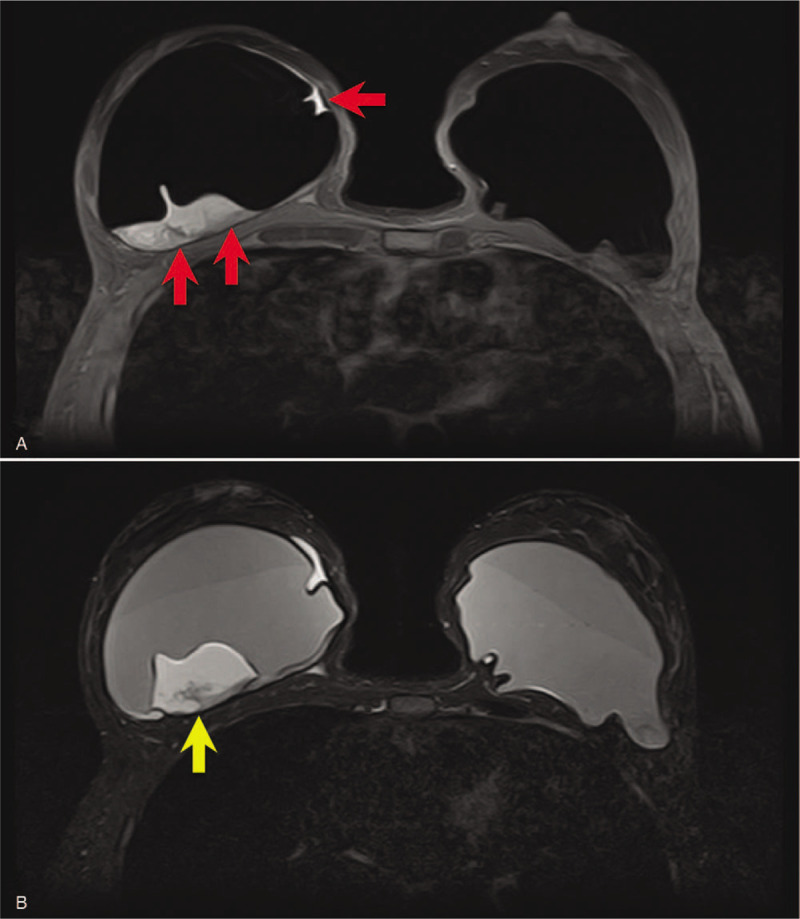
(A) Breast MR confirms the presence of a large periprosthetic seroma visible on the right sinus (red arrows). (B) In the corresponding T2-mode MR, amorphous material adhered to the back of the right prosthesis and due to fibrin deposits (yellow arrow) is recognizable.

In 2007, a 55-year-old Native America immigrant woman, from an indigenous tribe of Chickasaw (Oklahoma), underwent breast augmentation for esthetic purposes. Ten years later, in November 2017, the patient was referred to our Institute with clear asymmetry of the breasts due to an increase in volume of the right breast (Fig. 1). Mammography showed a double contour at the level of the right prosthesis (Fig. 2A) in comparison with the left prosthesis (Fig. 2B) and compatible with periprosthetic effusion: this corresponds to the external profile of the capsule (yellow arrows) and of the prosthesis (red arrows) which enclose the seroma, which is less radiopaque than the prosthesis. An ultrasound examination confirmed an abundant periprosthetic seroma that was drained and 200 mL of dark brown liquid was extracted by ultrasound-guided needle aspiration for cytological analysis. Furthermore, axillary, supraclavicular, and inguinal lymphadenopaties were not detected. Breast magnetic resonance (MR) confirmed a large periprosthetic fluid collection up to 5 cm in thickness that enveloped and compressed the prosthesis (Fig. 3A). The thickened, irregular, and hyperemic right-hand capsule in the anterior-superior portions showed multiple tiny nonvascularized solid aggregates adhered to the prosthesis in the internal portion, attributable to fibrin deposits and amorphous material (Fig. 3B); we reported this extremely particular and anomalous finding contained inside a periprosthetic seroma, many years after surgery, after excluding a previous trauma.

The cytological analysis of the exudate collected showed amorphous acidophilic material incorporating lymphocytes and foamy histiocytes and a number of large-sized atypical cells with irregular nuclei with CD30 immunoreactivity, leucocyte common antigen (CD45) compatible with BI-ALCL. This diagnosis was confirmed by histological examination following capsulectomy with the removal of the subglandular texturized prosthetic implants: the malignant cells were confined to the intracapsular fluid and to an internal layer of fibrinous tissue which adhered abundantly to the inner surface of the capsule where necrotic debris was found, exactly as described in MR. The final oncological stage attributed to this patient was T1N0M0, stage IA according to the TNM staging system.^[5]^ For this reason, the patient did not undergo chemotherapy or radiotherapy and, currently, >24 months after surgery, she is in follow-up with no signs of recurrent disease. The patient provided informed consent for all diagnostic and surgical procedures and for scientific dissemination.

## 3 Discussion

The pathogenetic mechanism of BI-ALCL is based on a chronic inflammation due to the presence of a prosthesis and the degradation products deriving from it, often sustained by trauma, genetic predisposition, immunological alterations, or bacterial infections^[3,6–8]^; it does not seem to correlate significantly with the type of implant material (silicone, hydrogel, saline), surgery (oncological or aesthetic), or position of the prosthesis (subglandular, submuscular), whereas a greater correlation with rough-surface implants (ie, texturized) has been previously described as in our case.^[4,6–9]^

The disease appears in about 2two-thirds of cases as a form affecting only the prosthetic capsule and in the remaining cases as an infiltrative form associated with mammary neoplastic mass or lymph node involvement and systemic extension.^[6,10]^ The 2 forms described could represent different stages of the same disease with a very different prognosis: a favorable outcome in cases of disease limited to the prosthetic capsule with a >90% remission following a capsulectomy alone; on the contrary, a more aggressive outcome is generally observed in advanced stages.^[9–12]^ An incomplete resection, an associated mass, or lymph node involvement will require adjuvant treatment such as chemotherapy or radiation therapy. There is no need for a radical mastectomy or a sentinel node biopsy, and full axillary dissection is reserved only for multinode metastasis. In this case, the tumor staging system T1 means that the lymphoma cells was confined to the luminal surface and a complete capsulectomy conferred an excellent prognosis. This is why, an early diagnosis is of fundamental importance, but it is also difficult due to lack of knowledge and the nonunivocal signs of disease, which often do not allow the identification of early pathognomonic radiological signs. The clinical onset is generally subtle, consisting in most cases (48%–70%) of an abundant and persistent periprosthetic effusion occurring many years after surgery. The median interval time between breast implant surgery and BI-ALCL diagnosis is 9 years.^[13]^ Other clinical signs are nonspecific or characterize advanced stages of disease: palpable masses (17%–31%), pain (21%), skin reddening (14%), capsular contracture (7%), skin lesions (7%), and fever (7%).^[6]^ The mammogram highlights the periprosthetic effusion as a “double contour,” showing a 73% sensitivity and a 50% specificity in detecting abnormalities, but is unable to distinguish between effusion and mass.^[12]^ Ultrasound (US) shows a sensitivity/specificity for effusion and a detection rate for masses of 75% to 84% and 46% respectively; MR Imaging of 33% to 82% and 50%, computed tomography (CT) of 55/83% and 50%, positron emission tomography with computed tomoraphy 38/83% and 64%.^[10]^

The radiological signs are also often ambiguous, highlighting the periprosthetic effusion or, in the most advanced forms, the breast or lymph node masses by ultrasound examination and breast MR with varying sensitivity and specificity.^[10]^

In our case, the cytological analysis of the fluid collection revealed atypical cells positive for CD30 immunocytochemistry with characterization of T–cell establishing a diagnosis of BI–ALCL in line with the most recent NCCN (National Comprehensive Cancer Network) guidelines^[14]^ and pathological examination of the prosthetic capsule showed a BI–ALCL without capsular invasion with the presence of granulation tissue, fibrin, and hemoglobin degradation products, retrospectively visible in breast MR: this is consistent with the mechanism of chronic inflammation underlying the process, although in literature it has never been described as a radiological sign. Where these findings to be confirmed by wider research, they could represent a potential early indicator of disease considering the pathogenic mechanism recognized as the basis of this oncological process, even considering as unlikely the possible correlation with surgery after 10 years. A further aid to diagnosis could also come from computer systems, known as CADs, from fusion MR/US imaging^[15]^ and from reporting systems based on radiomics, currently also being developed in senology.^[16–21]^

## Author contributions

**Conceptualization:** Daniele La Forgia, Annamaria Catino, Alfonso Fausto.

**Data curation:** Daniele La Forgia, Daniela Cutrignelli, Arianna Maiorella, Cosmo Ressa, Anna Scattone.

**Formal analysis:** Daniele La Forgia, Gianluca Gatta, Marco Moschetta, Alfonso Fausto.

**Methodology:** Marco Moschetta, Liliana Losurdo, Aurelio Portincasa.

**Resources:** Daniele La Forgia, Arianna Maiorella, Cosmo Ressa, Anna Scattone.

**Supervision:** Aurelio Portincasa, Cosmo Ressa, Liliana Losurdo, Marco Moschetta.

**Writing—original draft:** Daniele La Forgia, Liliana Losurdo, Daniela Cutrignelli, Annamaria Catino, Alfonso Fausto, Aurelio Portincasa.

**Writing—review and editing:** Daniele La Forgia, Annamaria Catino, Alfonso Fausto, Arianna Maiorella, Gianluca Gatta, Anna Scattone, Daniela Cutrignelli, Liliana Losurdo, Anna Scattone.

All authors have read and agreed to the published version of the manuscript.
